# Supplementary material for: Fungal parasitism on diatoms alters formation and bio–physical properties of sinking aggregates
Source: Commun Biol. 2023 Feb 21;6:206. doi: 10.1038/s42003-023-04453-6 (PMC9944279; doi:10.1038/s42003-023-04453-6)
Supplement: Supplementary file 2 — Supplementary Materials [file 42003_2023_4453_MOESM2_ESM.pdf]

Supplementary Materials

Fungal parasitism on diatoms alters formation and bio–physical properties of sinking aggregates

Isabell Klawonn*, Silke Van den Wyngaert, Morten H. Iversen, Tim J.W. Walles, Clara M. Flintrop, Carolina Cisternas-Novoa, Jens C. Nejstgaard, Maiko Kagami, Hans-Peter Grossart

*Corresponding author. isabell.klawonn@io-warnemuende.de

This PDF file includes:

Supplementary Box S1
Supplementary Methods S1
Figures S1 to S5
Table S1
References

Other Supplementary Materials for this manuscript include:

Supplementary_Data_1 (.xlsx file)
Supplementary_Data_2 (.xlsx file)
Supplementary_Data_3 (.xlsx file)
Supplementary_Data_4 (.xlsx file)
Supplementary_Data_5 (.xlsx file)
Supplementary_Data_6 (.xlsx file)

Supplementary Box S1. Symbols, terms, and abbreviations used in the main text.

Symbol	Unit	Parameter
A	mm^2	Cross-sectional area (aggregate)
C_D	unitless	Drag coefficient (aggregate)
C-specific respiration	d^{-1}	Fractional carbon-specific respiration per day (aggregate)
C-specific respiration	% m^{-1}	Percent carbon-specific respiration per meter settled (aggregate)
d	mm	Diameter (aggregate), herein represented by the *ECD*
Δd	mm	Width of aggregate size class
D_3	unitless	Fractal dimension, three-dimensional (aggregate)
ECD	mm	Equivalent circular diameter (aggregate)
L	m^{-1}	Remineralization length scale
μ	d^{-1}	Growth rate of diatom culture during exponential growth
$N(d)$	# L^{-1}	Aggregate number concentration
$n(d)$	# L^{-1} mm^{-1}	Aggregate size spectrum
nVd	mm^3 L^{-1}	Aggregate volume spectrum
Re	unitless	Reynolds number (aggregate)
U	m d^{-1}	Settling velocity (aggregate), measured
V (or V_{agg})	mm^3	Volume (aggregate)
$\rho_{s\text{-}agg}$	g cm^{-3}	Mass density (aggregates)
$\Delta\rho_{s\text{-}agg}$	mg cm^{-3}	Excess density (aggregate)
$\rho_{s\text{-}cells}$	g cm^{-3}	Mass density (diatom cells)
φ	unitless	Porosity (aggregate)

Term/abbreviation	Explanation
Non-infected diatom	Healthy, intact cells with high levels of chlorophyll autofluorescence
Early-infected diatom	*Synedra* with encysted zoospore (3 µm)
Maturely-infected diatom	*Synedra* with mature sporangium (7.5 µm)
Post-infected diatom	*Synedra* with empty sporangium (open operculum), remains of chitinous cell walls as a sign of previous infections after zoospore discharge
Decaying diatom	Empty silica frustule with no chlorophyll autofluorescence, no signs of previous infections (not involved in chytrid infections).
Non-inf	Non-infected treatment (without fungal microparasite)
Inf	Fungal-infected treatment (with fungal microparasite)
POC	Particulate organic carbon
DOC	Dissolved organic carbon
EPS	Extracellular Polymeric Substances
TEP	Transparent Exopolymer Particles
CSP	Coomassie Blue Stained Particles

Supplementary Methods

Supplementary Methods S1. TEP and CSP analyses

For spectrophotometric TEP determination, filters were placed in 15-mL Falcon tubes and 4 mL of extraction solution (80% H_2SO_4) were added [1,2]. Tubes were gently shaken for 2 h at room temperature. The absorbance of the extracted AB stain in solution was measured spectrophotometrically (787 nm). Concentrations of TEP are reported relative to a xanthan gum standard (µg XG equivalents L^{-1}). A 5-point calibration curve was prepared, covering the expected concentration range and yielding a strong linear correlation between absorbance and the mass of XG (R^2=0.97). For spectrophotometric CSP determination, filters were placed in 15 mL Falcon tubes and 4 mL of extraction solution (3% SDS in 50% isopropyl alcohol) were added. Tubes were gently shaken for 2 h, and additionally sonicated in a water bath (50–60 kHz) at 37°C. The absorbance of the extracted Coomassie Brilliant Blue G (CBB-G) stain was measured spectrophotometrically (615 nm). CSP concentrations are reported relative to a bovine serum albumin standard (µg BSA equivalents L^{-1}). A 4-point calibration curve was prepared following [1,3], ranging over the expected concentrations and yielding a strong linear correlation between absorbance and the mass of BSA (R^2=0.95).

Supplementary Figures

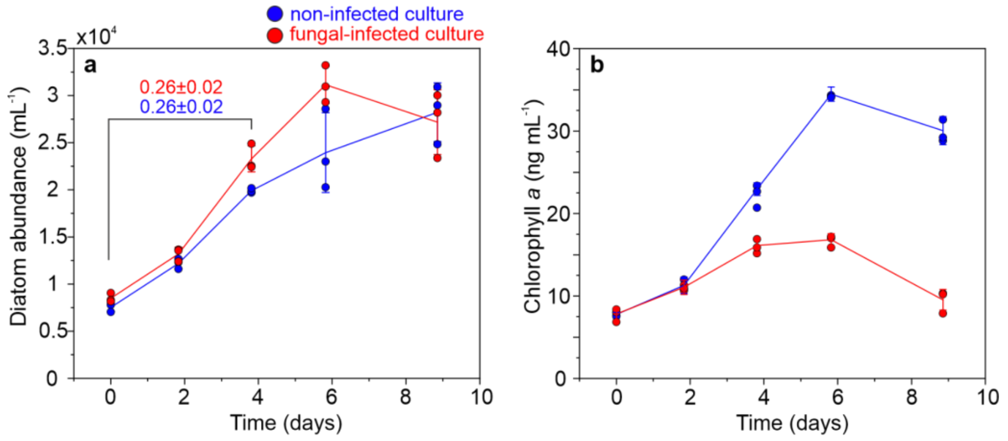

Supplementary Figure S1. *Synedra* abundance and chlorophyll *a* content in the diatom–fungi co-cultures. (a) *Synedra* cell abundances in the absence or presence of the fungal microparasite. *Synedra* counts include noninfected, infected and decaying cells. Growth rates are given for the period from day 0 to day 4 (mean±sd, *N*=3 incubation flasks). **(b)** For comparison, chlorophyll *a* contents are re-plotted from Figure 2c in the main document. Data in (**a, b**) are shown as single data points. Lines connect mean±SD values for consecutive time points (symbols for mean values are not displayed). Blue and red symbols/lines represent data from the non-infected and fungal-infected cultures, respectively.

Further explanation to Supplementary Figure S1

Growth rates were similar for noninfected and infected *Synedra* populations during exponential growth (day 0–4, 0.26±0.02 and 0.26±0.02 d^{-1}, respectively, *P*=0.64, *t*-test, *N*=3 incubation flasks). These growth rates compare well with previously reported rates of 0.24 d^{-1} and 0.22^{-1} for non-infected and infected *Synedra* cultures, respectively [4]. At 47% infection prevalence (day 6), *Synedra* abundance decreased, presumably due to mortality rates that exceeded growth rates. The initial abundances of healthy, noninfected *Synedra* cells on day 0 were similar in both culture treatments (7,460±460 and 7,497±205 cells mL^{-1} in the noninfected and infected culture, respectively, *P*=0.91). The higher total *Synedra* abundances in the fungal-infected culture in comparison to the noninfected culture on day 0 are explained by the added inoculum of infected cells. Those cells did not contribute to any further cell division since fungal infections are lethal to the diatom host cell. Growth rates *μ* of *Synedra* populations were calculated as

$$\mu = \frac{ln(N_{day\,4}) - ln(N_{day\,0})}{(t_{day\,4} - t_{day\,0})} \qquad \text{(Eq. S1)}$$

where $N_{day\ 4}$ and $N_{day\ 0}$ are the cell abundances (including all cell types: noninfected, infected, and decaying cells) at $t_{day\ 4}$ and $t_{day\ 0}$ [5].

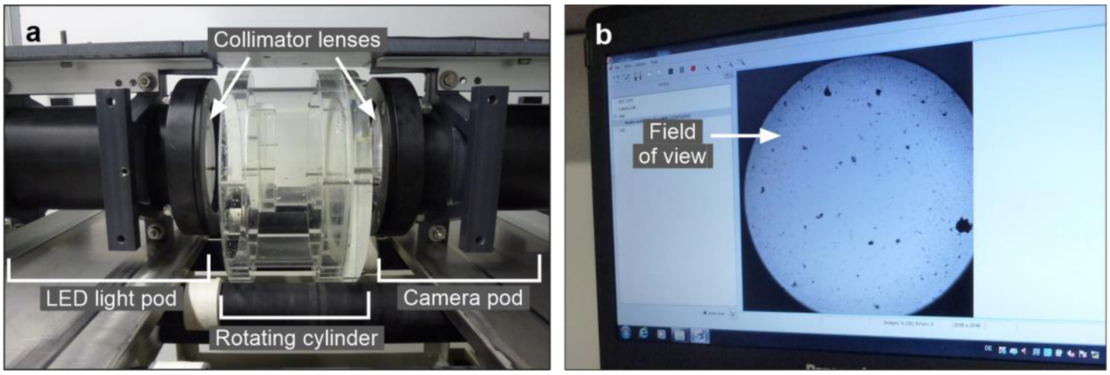

Supplementary Figure S2. Setup for monitoring the formation of aggregates in rotating cylindrical tanks using MDPI shadowgraph imaging. Each image captured a circular field of view (*d*=6.7 cm) along the entire cylinder depth (*l*=10 cm), equal to 0.35 L.

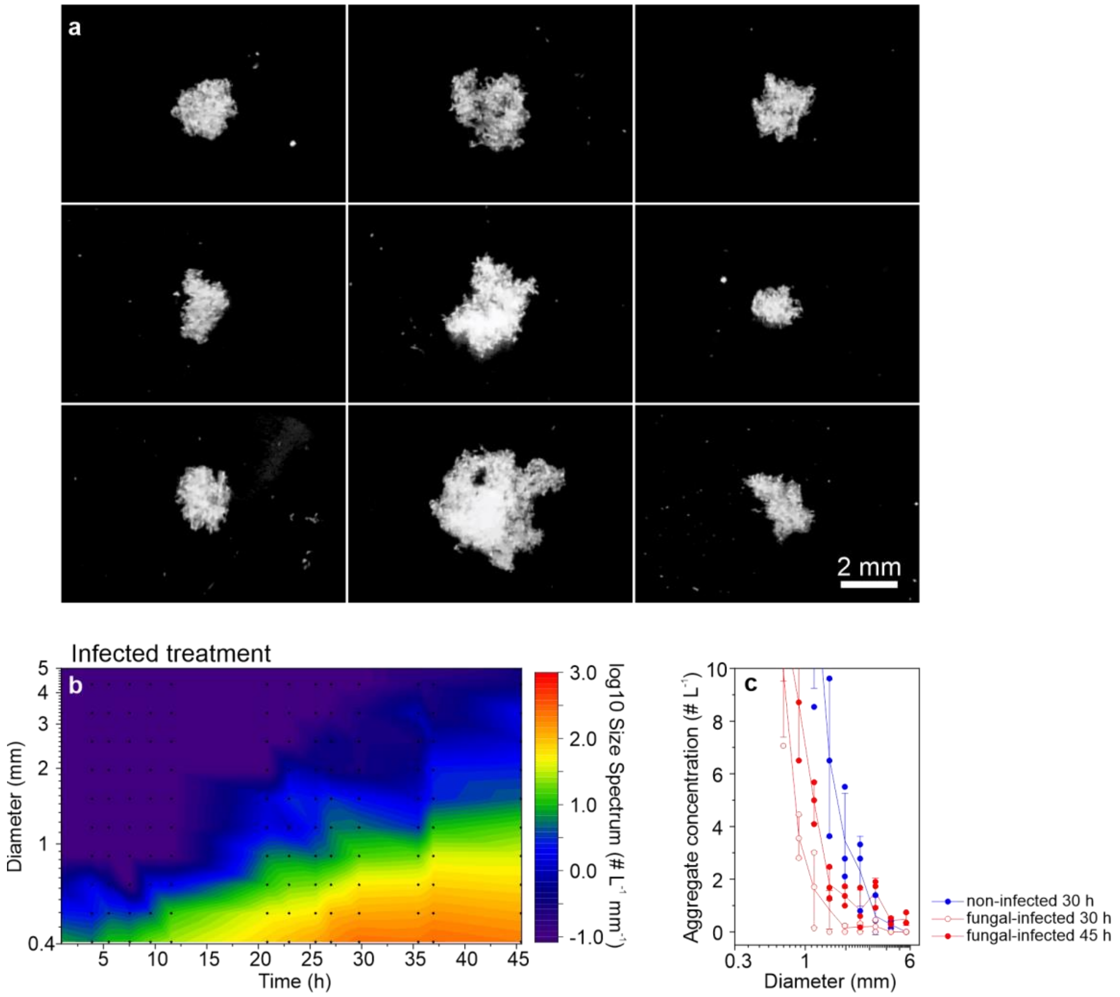

Supplementary Figure S3. Photographs of aggregates and aggregate formation from fungal-infected diatom cultures, recorded in rotating cylindrical tanks via imaging. (a) Exemplary photographs of similar-sized aggregates, which were picked individually after their formation in rotating cylinders and used to analyze their physical and biological properties (Table 1 in the main document). **(b)** Size spectra of aggregates, showing the number of aggregates per volume normalized to the width of each size bin. Log-transformation was used to visualize the wide range of size spectra. Values of -1.0 indicate that no aggregates in the respective size bin were present. **(c)** Aggregate concentration after 30 and 45 h. Since 1–5 mm-large aggregates were low in abundance after 30 h in the fungal-infected populations, the aggregate formation was extended to 45 h for the infected treatment, to be able to sample similar-sized aggregates in sufficient numbers from both treatments. Data in (**c**) are shown as single data points (triplicates) and lines connect mean values for consecutive time points (symbols for mean values are not displayed, but standard deviations of mean values are displayed as error bars). Blue and red items represent data from the non-infected and infected cultures, respectively.

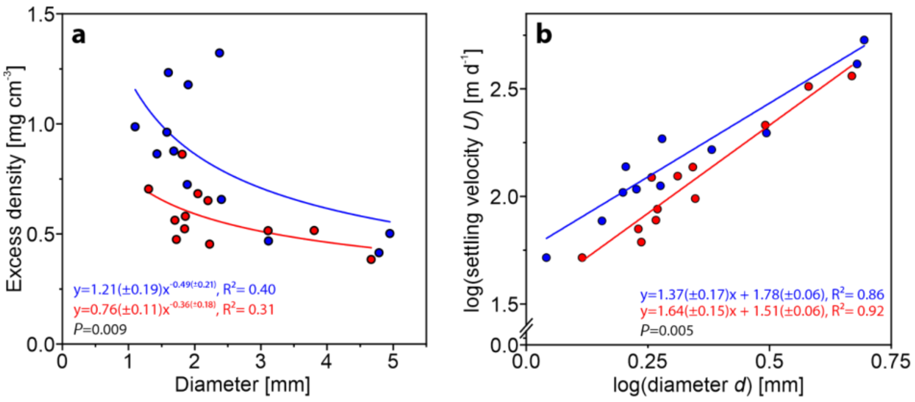

Supplementary Figure S4. Physical properties of aggregates plotted against the aggregate size of non-infected and infected aggregates, which were formed in rotating cylinders. (a) Size~excess density relationship, depicted by a power function-based curve fit, according to [6]. **(b)** Linear curve fit of aggregate sizes (diameter) and settling velocity after log–log transformation. The slope of d and U after log–log transformation was used to derive the fractal dimension D_3 ($\boldsymbol{U \sim d^{D_3-1}}$). (**a, b**) P-values indicate statistically significant differences between both treatments, comparing the curve fits. (**a, b**) Blue and red items represent data from the non-infected and infected cultures, respectively.

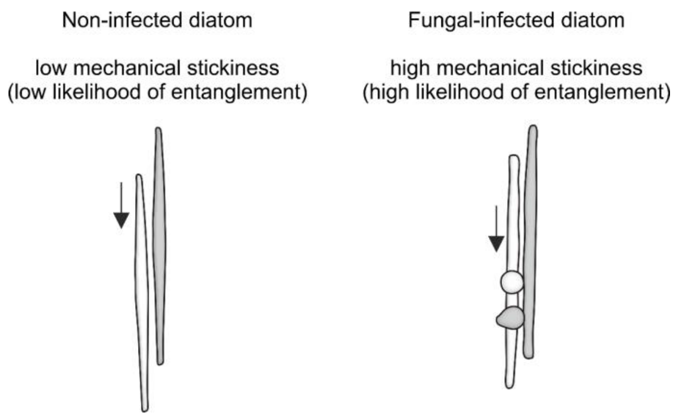

Supplementary Figure S5. Illustration of mechanical stickiness. The term "mechanical" stickiness was used by Kiørboe, et al. [7] who wrote: *"Many phytoplankters, including two of the diatoms studied here (Thalassiosira pseudonana and Skeletonema costatum), have extruding spines or hairs that may give rise to "mechanical" sticking or entangling of particles."* Following this statement, we suggest that fungal sporangia on the outside of infected *Synedra* cells increased the effective cell size and, at the same time, the likelihood that two *Synedra* cells may entangle due to their appendages (*i.e.*, sporangia), as illustrated.

Supplementary Tables

Supplementary Table S1. Recipe CHU-10 medium.

Supplement	Stock Solution* (g L^{-1})	mL L^{-1}	µmol L^{-1} in final medium
Na_2SiO_3 ($5H_2O$)	5.8	1	27
$Ca(NO_3)_2$ ($4H_2O$)	57.56	1	244
K_2HPO_4 ($3H_2O$)	8.5	1	37
$MgSO_4$ ($7H_2O$)	25	1	101
Na_2CO_3	20	1	189
add after autoclaving			
Fe-EDTA†	37	1	108
f/2 Vitamins‡	see below	1	

* stock solutions stored at RT
† filtered through a 0.2 µm membrane filter (stored at 4°C)
‡ sterile-filtered F/2 vitamins (stored at 4°C)

f/2 Vitamins

Supplement	Stock Solution mg mL^{-1}	/100ml H_2O	nmol L^{-1} in final medium
Vitamin B12	5 mg 5 mL^{-1} DI H2O	0.1 ml	0.738
Biotin	1 mg 10 mL^{-1} DI H2O	1.0 ml	4.1
Thiamine HCl		20 mg	593

Further chemicals:

Lugol solution: 10 g potassium iodide (KI), 70 mL distilled water, 5 g iodide (I_2), and 3 g sodium acetate

References

1. Passow, U. & Alldredge, A. L. A dye-binding assay for the spectrophotometric measurement of transparent exopolymer particles (TEP). *Limnol. Oceanogr.* **40**, 1326-1335, doi:10.4319/lo.1995.40.7.1326 (1995).
2. Engel, A. in *Practical Guidelines for the Analysis of Seawater* (eds Wurl O & Raton B) (CRC Press, 2009).
3. Cisternas-Novoa, C., Lee, C. & Engel, A. A semi-quantitative spectrophotometric, dye-binding assay for determination of Coomassie Blue stainable particles. *Limnol. Oceanogr. Methods* **12**, 604-616, doi:10.4319/lom.2014.12.604 (2014).
4. Maier, M. & Peterson, T. Observations of a diatom chytrid parasite in the lower Columbia River. *Northwest Sci.* **88**, 234-245, doi:10.3955/046.088.0306 (2014).
5. Guillard, R. R. L. in *Handbook of phycological methods: Culture methods and growth measurement* (ed Janet Stein) Ch. 19, 289-311 (Cambridge University Press, 1973).
6. van der Jagt, H., Friese, C., Stuut, J.-B. W., Fischer, G. & Iversen, M. H. The ballasting effect of Saharan dust deposition on aggregate dynamics and carbon export: Aggregation, settling, and scavenging potential of marine snow. *Limnol. Oceanogr.* **63**, 1386-1394, doi:10.1002/lno.10779 (2018).
7. Kiørboe, T., Andersen, K. P. & Dam, H. G. Coagulation efficiency and aggregate formation in marine phytoplankton. *Mar. Biol.* **107**, 235-245, doi:10.1007/BF01319822 (1990).
