# Supplementary material for: Fungal parasitism on diatoms alters formation and bio–physical properties of sinking aggregates
Source: Commun Biol. 2023 Feb 21;6:206. doi: 10.1038/s42003-023-04453-6 (PMC9944279; doi:10.1038/s42003-023-04453-6)
Supplement: Supplementary file 3 — Description of Additional Supplementary Files [file 42003_2023_4453_MOESM3_ESM.pdf]

Description of Additional Supplementary Files

File name: Supplementary Data 1
Description: Source data for Fig. 2.

File name: Supplementary Data 2
Description: Source data for Fig. 3.

File name: Supplementary Data 3
Description: Source data for Fig. 4.

File name: Supplementary Data 4
Description: Source data for Fig. 5.

File name: Supplementary Data 5
Description: Source data for Fig. 6.

File name: Supplementary Data 6
Description: Source data for Fig. S1–S3, Data microscopically-derived TEP/CSP.
