# Supplementary material for: Fungal parasitism on diatoms alters formation and bio–physical properties of sinking aggregates
Source: Commun Biol. 2023 Feb 21;6:206. doi: 10.1038/s42003-023-04453-6 (PMC9944279; doi:10.1038/s42003-023-04453-6)
Supplement: Supplementary file 10 — Reporting Summary [file 42003_2023_4453_MOESM10_ESM.pdf]

nature portfolio

Corresponding author(s): Isabell Klawonn

Last updated by author(s): Dec 16, 2022

Reporting Summary

Nature Portfolio wishes to improve the reproducibility of the work that we publish. This form provides structure for consistency and transparency in reporting. For further information on Nature Portfolio policies, see our Editorial Policies and the Editorial Policy Checklist.

Statistics

For all statistical analyses, confirm that the following items are present in the figure legend, table legend, main text, or Methods section.

n/a	Confirmed	
☐	☒	The exact sample size (n) for each experimental group/condition, given as a discrete number and unit of measurement
☐	☒	A statement on whether measurements were taken from distinct samples or whether the same sample was measured repeatedly
☐	☒	The statistical test(s) used AND whether they are one- or two-sided<br>*Only common tests should be described solely by name; describe more complex techniques in the Methods section.*
☒	☐	A description of all covariates tested
☐	☒	A description of any assumptions or corrections, such as tests of normality and adjustment for multiple comparisons
☐	☒	A full description of the statistical parameters including central tendency (e.g. means) or other basic estimates (e.g. regression coefficient) AND variation (e.g. standard deviation) or associated estimates of uncertainty (e.g. confidence intervals)
☐	☒	For null hypothesis testing, the test statistic (e.g. F, t, r) with confidence intervals, effect sizes, degrees of freedom and P value noted<br>*Give P values as exact values whenever suitable.*
☒	☐	For Bayesian analysis, information on the choice of priors and Markov chain Monte Carlo settings
☒	☐	For hierarchical and complex designs, identification of the appropriate level for tests and full reporting of outcomes
☒	☐	Estimates of effect sizes (e.g. Cohen's d, Pearson's r), indicating how they were calculated

Our web collection on statistics for biologists contains articles on many of the points above.

Software and code

Policy information about availability of computer code

Data collection	ImageJ 1.51p
Data analysis	Excel 2016, R 3.3.0, Origin2021, ImageJ 1.51p

For manuscripts utilizing custom algorithms or software that are central to the research but not yet described in published literature, software must be made available to editors and reviewers. We strongly encourage code deposition in a community repository (e.g. GitHub). See the Nature Portfolio guidelines for submitting code & software for further information.

Data

Policy information about availability of data

All manuscripts must include a data availability statement. This statement should provide the following information, where applicable:

- Accession codes, unique identifiers, or web links for publicly available datasets
- A description of any restrictions on data availability
- For clinical datasets or third party data, please ensure that the statement adheres to our policy

All data are available in the main text and supplementary materials. Images used for particle analyses and the image processing code are uploaded on Dryad.

Human research participants

Policy information about studies involving human research participants and Sex and Gender in Research.

Reporting on sex and gender	n/a
Population characteristics	n/a
Recruitment	n/a
Ethics oversight	n/a

Note that full information on the approval of the study protocol must also be provided in the manuscript.

Field-specific reporting

Please select the one below that is the best fit for your research. If you are not sure, read the appropriate sections before making your selection.

☐ Life sciences ☐ Behavioural & social sciences ☒ Ecological, evolutionary & environmental sciences

For a reference copy of the document with all sections, see nature.com/documents/nr-reporting-summary-flat.pdf

Ecological, evolutionary & environmental sciences study design

All studies must disclose on these points even when the disclosure is negative.

Study description	Laboratory study with model system, combined with field-sampling and laboratory incubations. Treatment factor: infected vs. noninfected, factorial study design, four replicates
Research sample	Model system (pelagic diatom Synedra, parasitic chytrid, and bacteria). Field-sampled populations (Planktothrix, Synedra, and Fragilaria).
Sampling strategy	Intermittent sampling over time. Samples size was chosen as a compromise of experimental feasibility and statistical power.
Data collection	All data were recorded by the authors.
Timing and spatial scale	Laboratory part: April 2017, Field sampling April 18th, 2018
Data exclusions	No data were excluded.
Reproducibility	Pre-experiments were undertaken in a similar manner as the final experiment. The findings of the pre-experiments were coherent with the final data.
Randomization	Samples were allocated into groups, based on their replication and time point. The following order was used: sample 1 from time point 1, sample 1 from time point 2, ..., sample 2 from time point 1, sample 2 from time point 2, ...
Blinding	n/a

Did the study involve field work? ☒ Yes ☐ No

Field work, collection and transport

Field conditions	Water temperature 7.5°C, calm weather (no precipitation).
Location	53°08'33.8"N 13°01'38.0"E, water depth ca. 10 m
Access & import/export	The sampling station was ca. 100 m off the shore in a lake (Lake Stechlin, Germany). Access was granted with a rowing boat.
Disturbance	n/a

Reporting for specific materials, systems and methods

We require information from authors about some types of materials, experimental systems and methods used in many studies. Here, indicate whether each material, system or method listed is relevant to your study. If you are not sure if a list item applies to your research, read the appropriate section before selecting a response.

Materials & experimental systems

n/a	Involved in the study
☒	☐ Antibodies
☐	☒ Eukaryotic cell lines
☒	☐ Palaeontology and archaeology
☒	☐ Animals and other organisms
☒	☐ Clinical data
☒	☐ Dual use research of concern

Methods

n/a	Involved in the study
☒	☐ ChIP-seq
☒	☐ Flow cytometry
☒	☐ MRI-based neuroimaging

Eukaryotic cell lines

Policy information about cell lines and Sex and Gender in Research

Cell line source(s)	Own (new) isolate.
Authentication	n/a
Mycoplasma contamination	n/a
Commonly misidentified lines (See ICLAC register)	n/a
